# The Islamic faith and best interests

**DOI:** 10.1192/bjb.2019.4

**Published:** 2019-08

**Authors:** Jessica Two, Martin Curtice

**Affiliations:** Worcestershire Health and Care NHS Trust, New Haven Unit, Princess of Wales Community Hospital, Bromsgrove, UK

**Keywords:** Mental Capacity Act 2005, best interests, consent and capacity, psychiatry and law, transcultural psychiatry

## Abstract

This article reviews a 2017 Court of Protection case which assessed and decided issues relating to the Islamic faith and the Mental Capacity Act 2005. The case involved a 39-year-old Muslim man with learning difficulties. It centred on his ability to make decisions about two specific aspects of his faith – capacity for fasting and for the removal of pubic and axillary hair. The judgment describes how s.4 of the Act was applied in deciding these decisions under the doctrine of best interests. In doing so, it elucidates key principles which can be applied to similar cases of this and other faiths.

**Declaration of interest:**

None.

This article reviews the Court of Protection judgment *IH (Observance of Muslim Practice)* [2017].^1^ The case involved a 39-year-old man, IH. It was an influential case as it considered the observance of Muslim custom and practice in patients who lack capacity under the Mental Capacity Act 2005^2^ (MCA) and Islamic law. In reviewing the case, this article aims to bring out salient principles that may be of assistance to clinicians and services that have a responsibility for Muslim adults who lack capacity.

## Background to the case

IH suffered from a profound learning disability, with the intellectual developmental level of a 1–3-year-old, and had a diagnosis of atypical autism. For the first 35 years of his life he resided in his family home, where he was principally cared for by his father, TH. IH was born in Pakistan but had lived in West Yorkshire all his life. His family, devout Sunni Muslims, engaged in all daily activities associated with the Five Pillars of Islam – faith, prayer, charity, fasting, and pilgrimage – as well as fasting during Ramadan and celebrating Eid (the Five Pillars being the foundation of Muslim life and regarded as obligatory for Muslims). While at home, IH would participate in some of these religious practices as he was able. This included daily prayers and, earlier in his life, attending Mosque, although he eventually stopped attending as his behaviour was occasionally disruptive to others. TH, being a devout Muslim, was motivated to ensure his son was supported, as was practical, to adhere as closely as possible to the Five Pillars of Islam.

Despite increased levels of support for the family, IH eventually moved into supported housing provided by the Local Authority. He required 2:1 support 24 hours a day, and was dependent on carers for all aspects of his care. His family visited regularly and he returned to the family home three times per week. IH experienced regular periods of agitation, which could occur ‘dramatically and without warning’. During these periods of agitation, which could be prolonged, IH had a tendency to kick, bite, punch, and/or spit at his carers, who had sustained injuries as a result.

The court was asked to consider two questions:

1) whether it was in IH's best interests to fast during the daylight hours of Ramadan; and
2) whether it was in his best interests for his axillary and pubic hair to be trimmed.

## The position of the parties involved in the application

TH contended that the removal of IH's pubic and axillary hair was a compulsory activity stipulated under Islamic law, and hence there was a ‘religious duty’ to observe this (albeit he modified this stance following expert evidence as below). His father used to shave his son when he was at home and in the first year when he moved into accommodation. TH held the view that having his son's pubic hair shaved was IH's ‘right’ and something he would have chosen to do himself had he the requisite capacity to do so. IH was provided with Halal food to eat and celebrated Eid with his family, but he had never been expected to fast during Ramadan as it was recognised that he would not understand the purpose of food and drink being withheld during daylight hours and that this might have caused him distress.

The local authority's position was that it recognised the importance of facilitating the religious observances of an individual, even those who lacked capacity. It was agreed that staff members would carry out the hair removal process every two weeks, although in actuality this did not happen, and IH had not had his pubic or axillary hair shaved for three years and never by his carers.

IH's social worker, herself a Muslim, expressed the view that Islam was a significant part of IH's identity and ‘something of which he should be proud’, and that ‘the observance of religious practices should be supported where possible to allow a person to continue to associate with their identity, background, culture and beliefs’. IH's main carer did not believe trimming of IH's pubic and axillary hair to be in his best interests, but the Team Manager of the Community Team for Learning Disabilities considered that, ‘on a fine balance’, it was.

While the court accepted that it was important to support the religious practices of IH, it concluded that the social worker ‘had significantly misunderstood IH's capacity to understand and reflect the concepts associated with the religion’ and that IH ‘has *no* understanding of the concept of Islam … he has *no* capacity to feel ‘proud’ of his religious heritage; nor is he able to actively ‘associate with’ an identity or culture …’.

## Capacity for fasting

There was no dispute among the parties that, according to the MCA, IH lacked capacity to make decisions regarding fasting during Ramadan. In relation to fasting, the judgment laid out six specific criteria that a person would be expected to understand in order to have capacity in this regard (Box 1). It was agreed that IH was not able to understand any of the six points listed, and that in view of the nature of his disability he would never acquire capacity to make these decisions and hence lacked capacity to make this decision.

Box 1 Specifics a person should be able to understand when making decisions regarding fasting.

1. What fasting is; the lack of food and liquid, eating and drinking.
2. The length of the fast.
3. If for religion, for custom (family or otherwise), for health-associated reasons or for other reasons.
4. If for religion reasons, which religion and why.
5. The effect of fasting on the body.
6. What the consequences would be of making a choice to fast and the risks of choosing to not fast or of postponing the decision.

## Capacity for removal of pubic and axillary hair

The specific aspects that a person would be expected to understand in order to have capacity to make decisions in regards to the trimming or removal of hair for religious or cultural reasons were elucidated by the court (Box 2). It was again agreed by all parties that IH did not have, and would never acquire, capacity in this regard.

Box 2 Specifics a person should be able to understand when making decisions regarding removal of hair for religious or cultural reasons.

1. Which parts of the hair are being removed – pubic, axillary, perianal, trunk, beard, leg, torso or head.
2. Whether the reason for the hair trimming/removal is religious, for the maintenance of good hygiene, custom or some other.
3. If for a religious reason, which religion and why.
4. What the consequences would be of making a choice to have hair trimmed/removed, and of not trimming/removing the hair.

## Requirements of the Islamic faith for those lacking capacity

When considering the degree to which those lacking capacity are expected to adhere to the Five Pillars of Islam, the court consulted Dr Ali, lecturer in Arabic and Islamic Studies at Cardiff University. Dr Ali advised that while certain actions under Islamic law are obligatory, allowances are made for those that are deemed to lack ‘legal competence’. This term was defined as ‘a capacity or a potential for mental functioning, required in a decision-specific manner, to understand and carry out decision-making.’ As with capacity under the MCA, capacity under Islamic law is always initially presumed to be present. A person can only be declared to be ‘legally incompetent’ by a court, following evidence from medical practitioners or experts in legal competence, whose opinions under Islamic law would be deemed ‘legitimate and authoritative’. A legally incompetent person (as well as terminally ill people, disabled people and minors) is deemed to be in a heightened state of spirituality and as such is exempt from adhering to many of the major rituals of Islam. All parties agreed that IH could be declared legally incompetent under Islamic law.

While fasting during the daylight hours of Ramadan is an obligatory custom for all legally competent Muslims, certain groups are exempt from fasting. These include minors, pregnant women, those who are travelling, the ill, and the incapacitous. Given that IH had been declared ‘legally incompetent’ under Islamic law, it was agreed that he met these exemption criteria and as such would not be expected to fast.

The holy book of Islam, the Qur'an, advises Muslims to uphold high standards of personal hygiene and to be ritually clean. As well as Quranic verses, there are teachings from Hadiths (traditions or sayings of the Prophet Muhammad revered as a major source of religious law and moral guidance second only to the authority of the Qur'an) advising Muslims as to the rulings of personal grooming. Under Islamic law, the cleaning of pubic or axillary hair is a religiously sanctioned practice deemed to be a normal human right (‘fitrah’) related to a pursuit for ritual purity and cleanliness. The removal of pubic and axillary hair is a recommended practice (‘mustahab’) but is not obligatory. While it would not be acceptable for a Muslim to expose his genitals, it is permissible for a Muslim who requires assistance with his care to have carers clean or shave his genitals. However, the removal of pubic and axillary hair is not a recommended practice for those that lack ‘legal competence’ and there is no suggestion that Islamic law requires IH's carers to carry out hair removal on his behalf or that his religious rights are being violated by their not doing so.

## Best interests decision-making

In determining what was in IH's best interests in relation to fasting during Ramadan and removal of his axillary and pubic hair, the court analysed Section 4 MCA – Best interests. This stipulates that, as far as is ‘reasonably ascertainable’, IH's past and present wishes and feelings (s.4(6)(a)), the beliefs that are likely to have influenced his decision had he had capacity (s.4(6)(b)), and the wishes of his family (s.4(7)(b)) should be taken into account. Explicit reference was made to the seminal Supreme Court judgment in *Aintree University Hospitals NHS Trust v James* [2013]^3^, which opined that any best interests test ‘should also contain a strong element of “substituted judgement”’, meaning that the person making the decision should place great emphasis on what the person is likely to have done had they had capacity to make the decision for themselves. However, while an important component, substituted judgement is not the entirety of a best interests decision. The Supreme Court further stated ‘The purpose of the best interests test is to consider matters from the patient's point of view. That is not to say his wishes must prevail, any more than those of a fully capable patient must prevail. We cannot always have what we want. Nor will it always be possible to ascertain what an incapable patient's wishes are’.

## Was it in IH's best interests to fast during Ramadan?

All parties agreed that IH would not be able to understand the purpose of withholding food or fluids from him during the daylight hours of Ramadan, and that to do so may cause him significant distress. There were also concerns that fasting might lead to mild dehydration, which could amplify side-effects of medication. The court concluded that it was not in IH's best interests to fast during Ramadan and granted a declaration that he should be relieved of this obligation.

## Was it in IH's best interests for his pubic and axillary hair to be trimmed?

The court noted that the local authority caring for IH had an obligation to ‘create a care environment and routine which is supportive of the religion’ of any person under their care and ‘to facilitate the person's access to, or observance of religious custom and ritual’. The court cited the provision of IH with a Halal diet, despite him having no understanding of the fact that the food he was provided with was Halal and no understanding of the significance of a Halal diet, as an example of the local authority recognising the need to respect IH's religion. However, in regards to the custom of shaving of IH's pubic and axillary hair, the court concluded that there was no obligation on the local authority to ensure that this religious custom was observed. The court's best interests reasoning on this is described in Box 3.

Box 3 Text from the judgment in reaching the conclusion that it was not in IH's best interests for his pubic and axillary hair to be trimmed

‘In short … there is simply no religious duty, or obligation on a person who lacks capacity (‘legal competence’ in Islam) to trim or shave his or her pubic and axillary hair, or on his carer to do so for them. IH does not need to acquire this state of ritual cleanliness in order to derive spiritual benefit as he already occupies an elevated status by virtue of his incapacity. Moreover, I am satisfied that IH himself derives no religious ‘benefit’ by having the procedure undertaken, as he would not understand its religious significance. It is of no consequence to me, in the consideration of these facts, that the carers may be blessed in the eyes of Islam in undertaking a ‘praiseworthy’ activity by trimming the hair; their interests are not my concern.’

‘I agree with TH … that if IH had capacity he probably would have observed this custom. … It would have been entirely consistent with the religious and cultural norm within his home and community. … However, this factor carries little weight in my overall reckoning given that in progressive Islamic religious teaching, as an incapacitous person IH is exempt from observing the Islam rituals because he is already on a heightened state of spirituality.’

This assessment also considered in depth the practical intricacies of staff attempting such a procedure under the best interests doctrine. The court was concerned that even if IH was cooperative, the procedure was likely to be anxiety-inducing and distressing for him, which could heighten his propensity to agitation and aggression. The court acknowledged that, while IH did not ‘have any sense of personal modesty’, the nature of such a procedure incurred ‘compromises to the preservation of dignity’.

## Conclusions of the court

The court concluded that it was not in IH's best interests to (1) fast during Ramadan, or (2) to have his pubic and axillary hair shaved in accordance with Islamic custom. The balancing act contained within the best interests assessment was eloquently described by the presiding judge as:

‘I have faithfully endeavoured to consider these issues from IH's point of view, while ultimately applying a best interests evaluation. IH has a life-long developmental condition and has never had the capacity to understand the tenets of Islam; the benefits of adherence to such rituals do not obtain for him, but for others. The fact is that by reason of his disability IH is absolved of the expectation of performing this recommended procedure, and there is no other clear benefit to him. The trimming of the pubic and axillary hair would serve no other purpose. I am anxious that IH should be spared additional stresses in his life, and wish to protect him and the staff from the risk of harm – an approach which itself has the endorsement of Islamic teaching'.

## Discussion

The judgment included consideration of Islamic bioethics in its decision-making and specifically that ‘No hurt no harm’ was a cardinal principle of this approach. The judgment opined that it would be wrong to create a situation whereby observance of Islamic custom could or would cause harm to the person or their carers. Islamic bioethics is an extension of Shariah (Islamic Law) and is intimately linked to the broad ethical teachings of the Qur'an.^4^ It teaches that the patient must be treated with respect and compassion, and that the physical, mental and spiritual dimensions of the illness experience should be taken into account. The principalist approach to biomedical ethics^5^ as a culturally sensitive approach is broadly accepted and has been discussed among Muslim scholars.^6^ The four general principles of this are: (1) respect for autonomy, (2) beneficence, (3) non-maleficence, and (4) justice. Justice is often regarded as being synonymous with fairness – it can be seen as the moral obligation to act on the basis of fair adjudication between competing claims.^4^ Gillon^7^ subdivided the obligations of justice into: (1) fair distribution of scarce resources (distributive justice); (2) respect for people's rights (rights based justice); and (3) respect for morally acceptable laws (legal justice). In this way, it can be seen that the MCA and the doctrine of best interests can readily be applied to individuals of the Islamic faith, and that Islamic bioethics underpins such an approach. Other legal cases involving various aspects of the Islamic faith include adoption,^8^ child care orders and immunisations,^9^ capacity to marry,^10^ capacity to marry and to have sexual relations^11^^,^^12^ and circumcision of a child.^13^

While this case centred on the Islamic faith, a similar approach can be taken in best interests cases for people of all faiths. Where needed, obtaining clarity from experts within a particular faith will be vital. Also, systematically applying the whole MCA rubric for s.4 Best interests decisions is of course paramount – the best interests checklist as advised by the MCA Code of Practice^14^ (Para 5.32) being vital for this. Within this assessment, it is important to establish a person's reasonably ascertainable past and present wishes and feelings, and the beliefs and values that would be likely to influence their decision if they had capacity (s.4(6)). Similarly, where practicable and appropriate, it is important to gain the views of significant others as to the decisions at hand (s.4(7)). While such collateral history can be invaluable, the intricacies of balancing and weighing such information can be complex, as was seen in a case involving the potential cessation of clinically assisted nutrition and hydration from an elderly Christian man with end-stage dementia.^15^ In needing to obtain history from the family for this best interests decision, the court noted that it was ‘important that the strength and conviction of their views is not allowed to detract from a steady appreciation of the welfare of the individual concerned’.

There has been a rapidly developing body of best interests case law in recent years emanating from the Court of Protection. This has included cases involving best interests decisions containing a significant religious or faith-based element (Box 4). The case of *Re BM*^17^ suggested an approach to the weighing up or balancing of elements within best interests decision-making. This approach, which could be applied as part of a balance sheet approach or used as free-standing, involved identifying ‘the factor of magnetic importance’ – this factor being the one that tips the balance and determines the eventual outcome. Although not finally accepted by the judge, counsel had proposed that it was BM's ‘very deep faith’ that was the magnetic factor in his case.

Box 4 Examples of best interests cases involving a religious or faith-based element

1. *Sandwell Metropolitan Borough Council v RG & Ors* [2013]^16^ – annulment of a marriage for a Sikh man with a learning disability.
2. *BM, Re* [2014]^17^ – the appointment of a deputy for property and financial affairs in a man of Christian faith who suffered an extensive cerebrovascular accident.
3. *The London Borough of Tower Hamlets v TB & Anor* [2014]^18^ – the assessment of where to live and capacity to consent to relations for a married Bangladeshi woman with moderate learning disabilities.
4. *P, Re (capacity to tithe inheritance)* [2014]^19^ – the capacity of a man with a chronic schizoaffective disorder to make a ‘tithe’ donation of 10% of an inheritance to a church.
5. *Wye Valley NHS Trust v B* [2015]^20^ – the potential amputation of a foot in a man with chronic schizophrenia in whom religiose delusions and auditory hallucinations had become so entrenched as to become an ‘intrinsic part of who he is’. This case has arguably been at the forefront of a sea change in how courts apportion weight to a person's views and beliefs as part of any best interests assessment.
6. *N, Re* [2015]^21^ – the determination of whether it was in the best interests for a Jewish woman with multiple sclerosis and in a minimally conscious state to continue to receive life-sustaining treatment by means of clinically assisted nutrition and hydration (CANH).

*The above judgments, and all within this article, can be found via www.bailii.org

The Law Commission review of Mental Capacity and Deprivation of Liberty law^22^ has recommended a legislative addition to s.4(6) MCA such that decision makers should ‘give particular weight to any wishes or feelings ascertained’. The Government response^23^ has accepted that this recommendation ‘should be enshrined in law’, noting that as part of a person-centred approach the principle of taking past and present wishes and feelings into account already represents good care practice.

## About the authors

**Jessica Two** is an ST6 and **Martin Curtice** is a Consultant in old age psychiatry at the Worcestershire Health and Care NHS Trust, New Haven Unit, Princess of Wales Community Hospital, Bromsgrove, UK.
